# Fusarium infection manifesting as toe cellulitis in HSCT recipient

**DOI:** 10.1002/jha2.382

**Published:** 2022-02-25

**Authors:** Sarah Malkiel, Or Kriger

**Affiliations:** ^1^ Pediatrics Sheba Medical Center Ramat Gan Israel

1


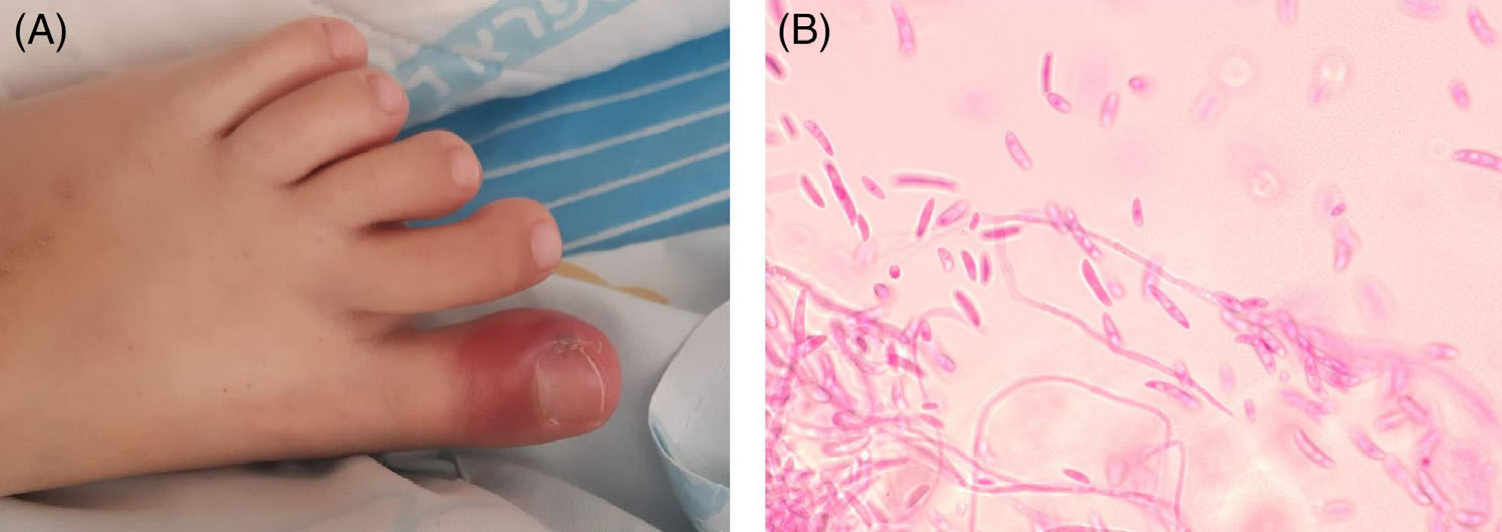


A 10‐year‐old girl with relapsed acute lymphoblastic leukemia underwent allogeneic hematopoietic stem cell transplantation. She was conditioned with total body irradiation, etoposide, and anti‐thymocyte globulin. On day +4, mild erythema and tenderness appeared on her left big toe. Broad spectrum antibiotics were administered. However, by day +7 the toe became dark red and extremely painful (Panel A), and the patient became febrile. A skin biopsy was performed and cultures yielded a mold fungus with hyaline septate hyphae on staining with Lactofuchsin phenol (Panel B), morphologically compatible with *Fusarium species*. Further molecular sequencing identified the mold as *Fusarium falciforme*. Blood cultures were negative, and a chest CT was normal. The patient was treated with amphotericin and isavuconazole. Of note, neutrophil engraftment occurred on day +13. The lesion improved gradually and resolved by day +30. *Fusarium* is commonly associated with onychomycosis, and can manifest as toe cellulitis in immunocompromised patients. Immune recovery plays a crucial role in clinical outcome.

